# Supplementary material for: Microthermal-induced subcellular-targeted protein damage in cells on plasmonic nanosilver-modified surfaces evokes a two-phase HSP-p97/VCP response
Source: Nat Commun. 2021 Jan 29;12:713. doi: 10.1038/s41467-021-20989-9 (PMC7846584; doi:10.1038/s41467-021-20989-9)
Supplement: Supplementary file 1 — Supplementary Information [file 41467_2021_20989_MOESM1_ESM.pdf]

Supplementary information for

Microthermal-induced subcellular-targeted protein damage in cells on plasmonic nanosilver-modified surfaces evokes a two-phase HSP-p97/VCP response

Martin Mistrik, Zdenek Skrott, Petr Muller, Ales Panacek, Lucie Hochvaldova, Katarina Chroma, Tereza Buchtova, Veronika Vandova, Libor Kvitek and Jiri Bartek

Correspondence to: Martin Mistrik (martin.mistrik@upol.cz) and Jiri Bartek (jb@cancer.dk)

Supplementary Figure 1

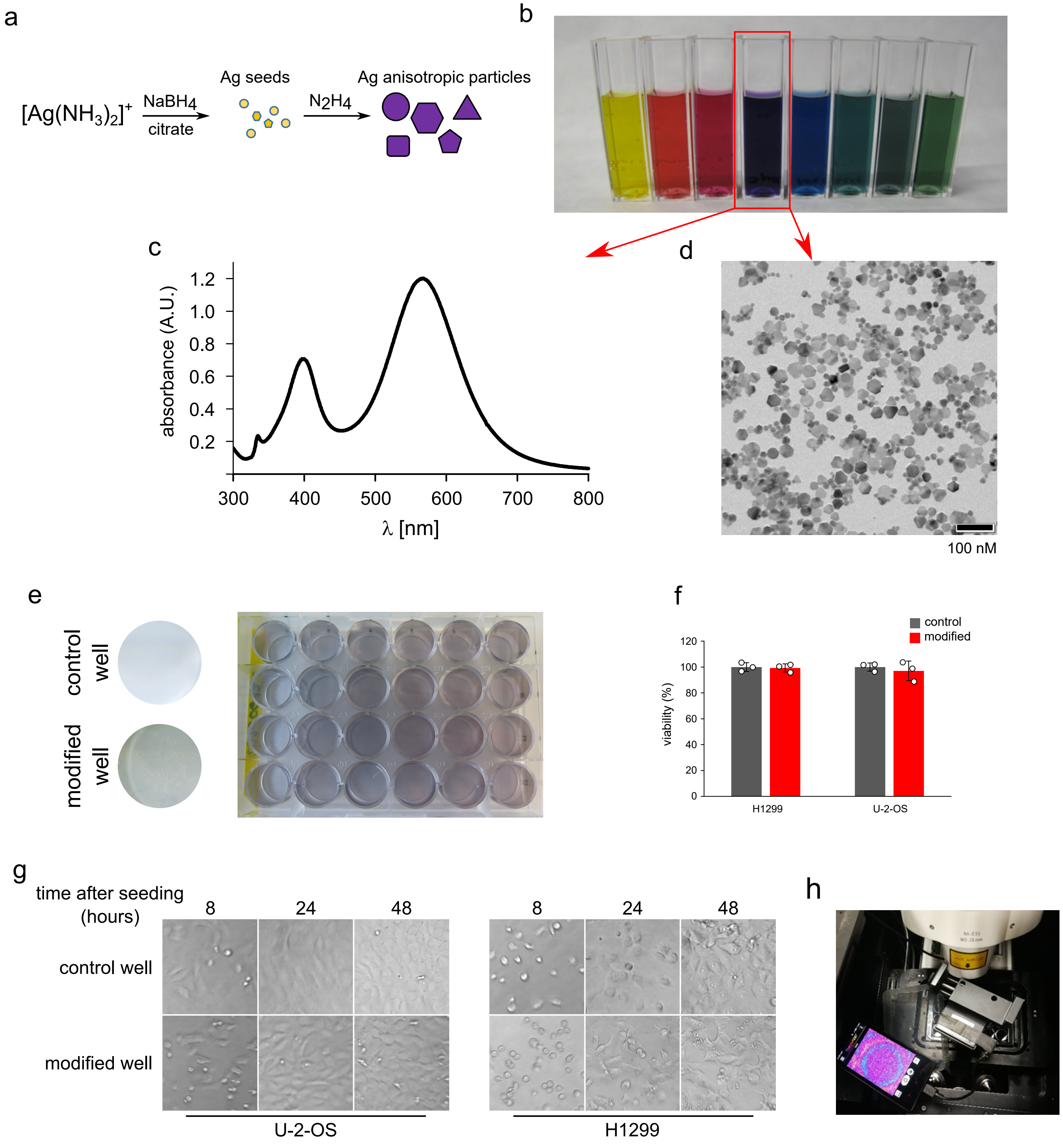

Supplementary Figure 1. Characterisation of plasmon-modified cultivation surface. a, Schematic representation of the synthesis of anisotropic silver nanoparticles. **b**, By tuning the conditions of silver NPs synthesis, the plasmon resonance frequency can be localized anywhere within the region of 440-740 nm, as reflected by various colors of the colloid dispersion. **c**, Absorption spectra of silver nanoparticles used in experimental setups in colloidal form. **d**, TEM micrograph of a colloidal suspension of anisotropic silver NPs. Representative results from three experiments. **e**, Photographs of the plasmon modified wells compared to the control plate. **f**, Cell viability assessment of cells grown on plasmon modified and control cell culture plates (mean, SD from 3 experiments). **g**, Photograph of adherence efficacy and morphological aspects of U-2-OS and H1299 cells seeded on control or modified wells showing no visible difference. Representative results from two experiments. **h**, Photograph of the thermal imaging setup inside the laser scanning microscope. Scale bar = 100 nm.

Supplementary Figure 2

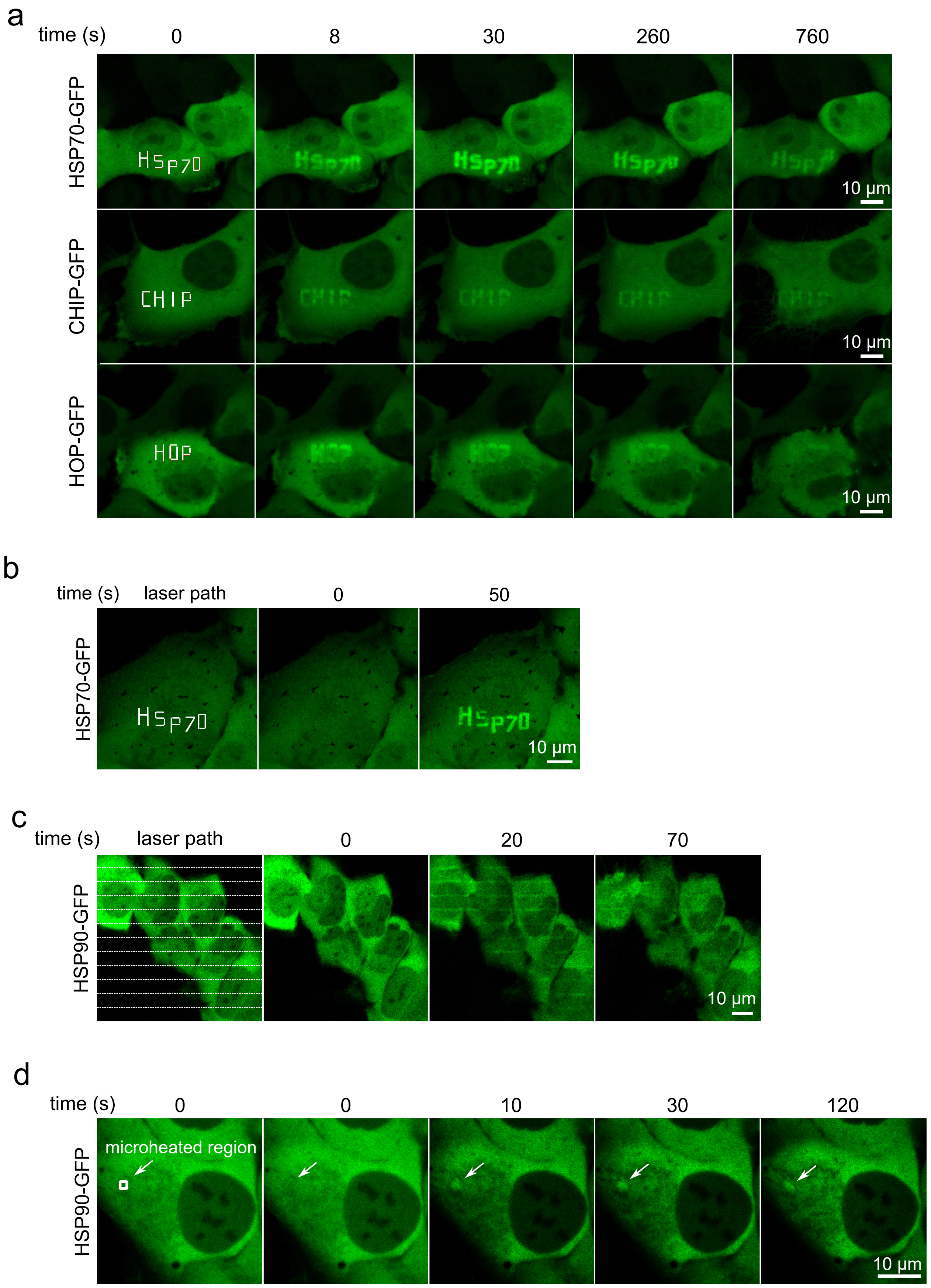

Supplementary Figure 2. Recruitment of various reporters to micro-heated regions. a, Recruitment of the three indicated GFP-tagged heat shock-related proteins to the micro-heated regions in H1299 cells grown on plasmon modified TPP plate. Microheated regions were exposed to 561 nm laser. The defined laser path is shown in white. Cells were followed in time. Representative results from two experiments. **b**, Recruitment of HSP70-GFP to the micro-heated region defined by laser path in U-2-OS cells grown on plasmon modified glass plate. Representative results from two experiments.**c**, U-2-OS cells grown on a plasmon modified Ibidi plate expressing HSP90-GFP were micro-heated by laser stripes (561 nm, 15% power) and followed in time, revealing transient recruitment of HSP90-GFP. Representative results from three experiments. **d**, Recruitment of HSP90-GFP to the micro-heated region in U-2-OS cells. Representative results from three experiments. Scale bars = 10 μm.

Supplementary Figure 3

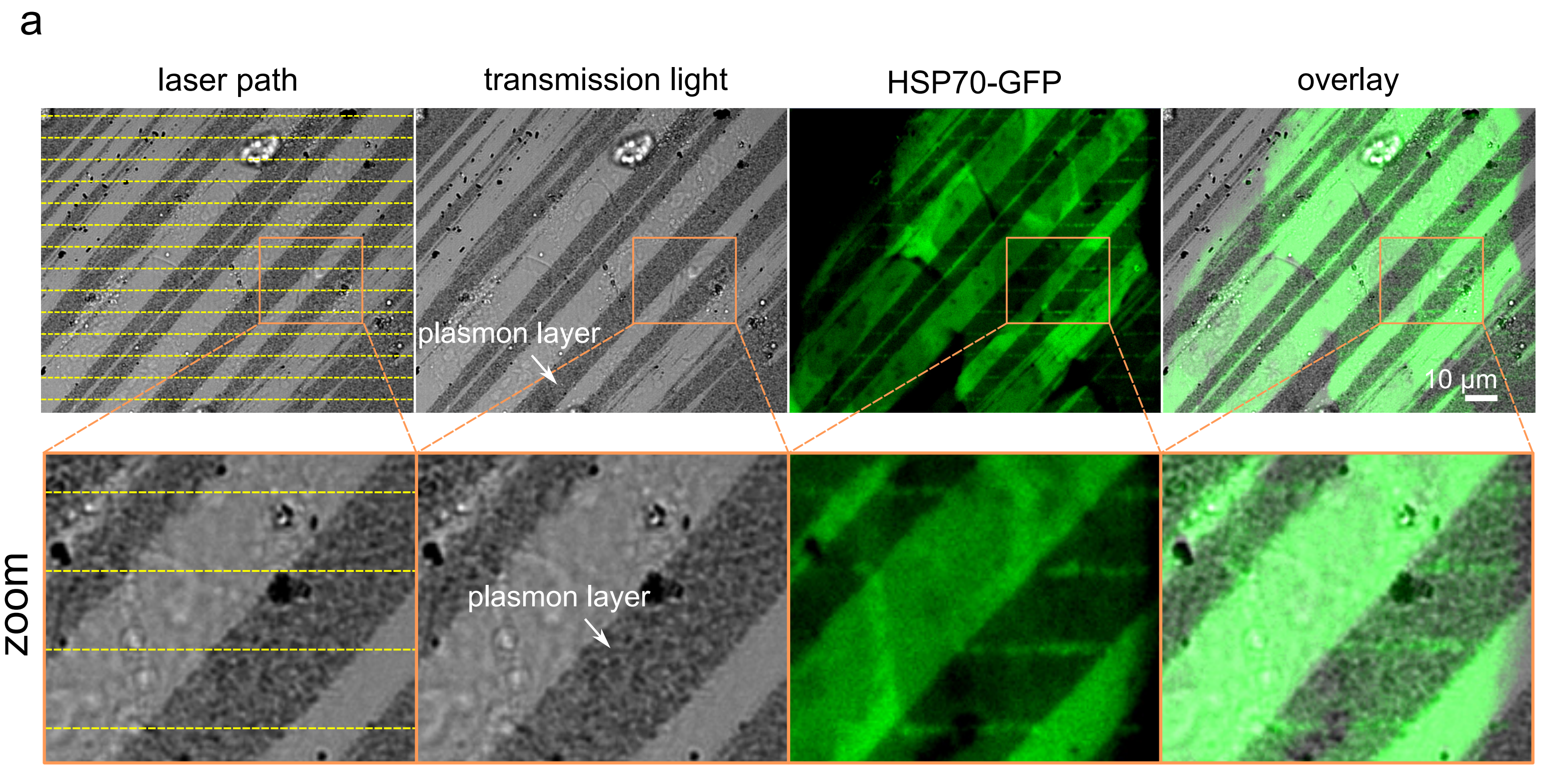

Supplementary Figure 3. Control experiment validating that the heat damage is plasmon layer-specific. a, Plasmon-modified well was first scratched by pipette tip to partially remove the plasmon layer and next HSP70-GFP expressing U-2-OS cells were seeded to the well. The presence or absence plasmon layer is well visible on the transmission light and also in the fluorescence mode as the plasmon layer is generally less transparent. Cells were micro-heated by collinear laser stripes. The micro-heat damage (documented by HSP70-GFP recruitment) is detectable only in the parts of the cells which were above the plasmon layer. Representative results from three experiments. Scale bar = 10 μm.

Supplementary Figure 4

a

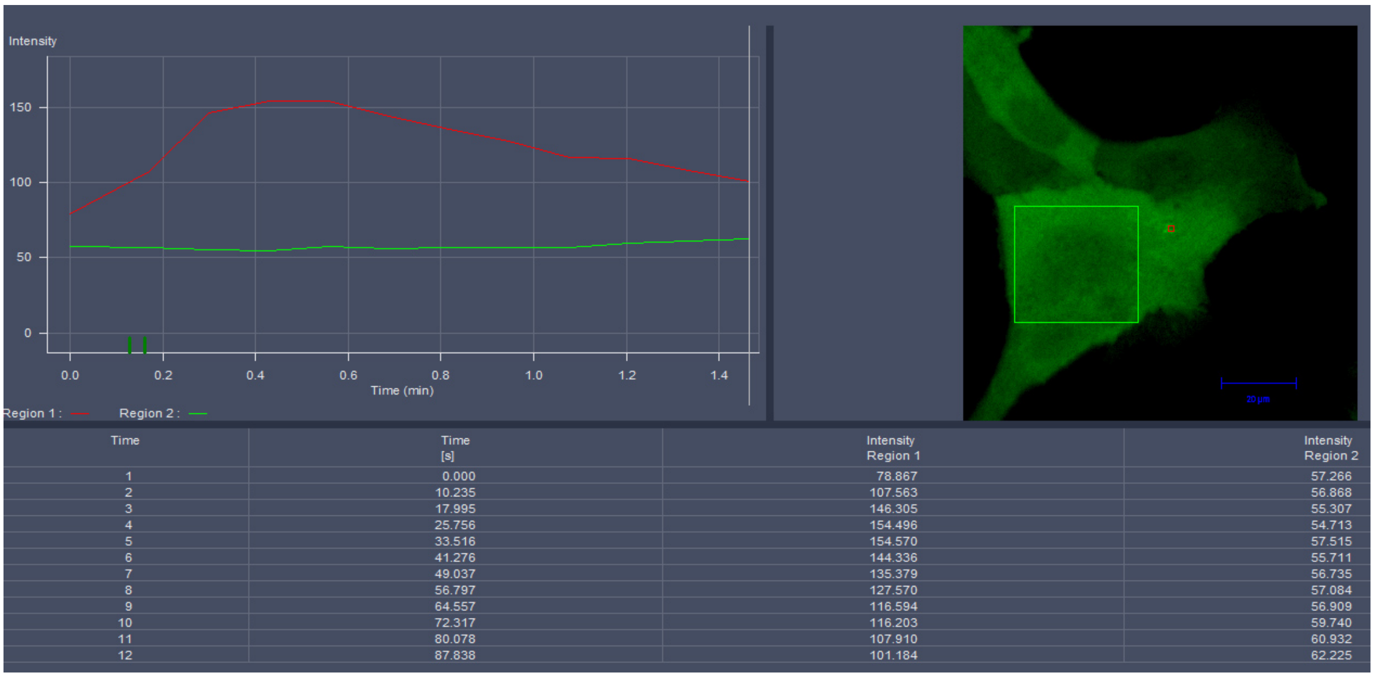

Time	Time [s]	Intensity Region 1	Intensity Region 2
1	0.000	78.867	57.266
2	10.235	107.563	56.868
3	17.995	146.305	55.307
4	25.756	154.496	54.713
5	33.516	154.570	57.515
6	41.276	144.336	55.711
7	49.037	135.379	56.735
8	56.797	127.570	57.084
9	64.557	116.594	56.909
10	72.317	116.203	59.740
11	80.078	107.910	60.932
12	87.838	101.184	62.225

b

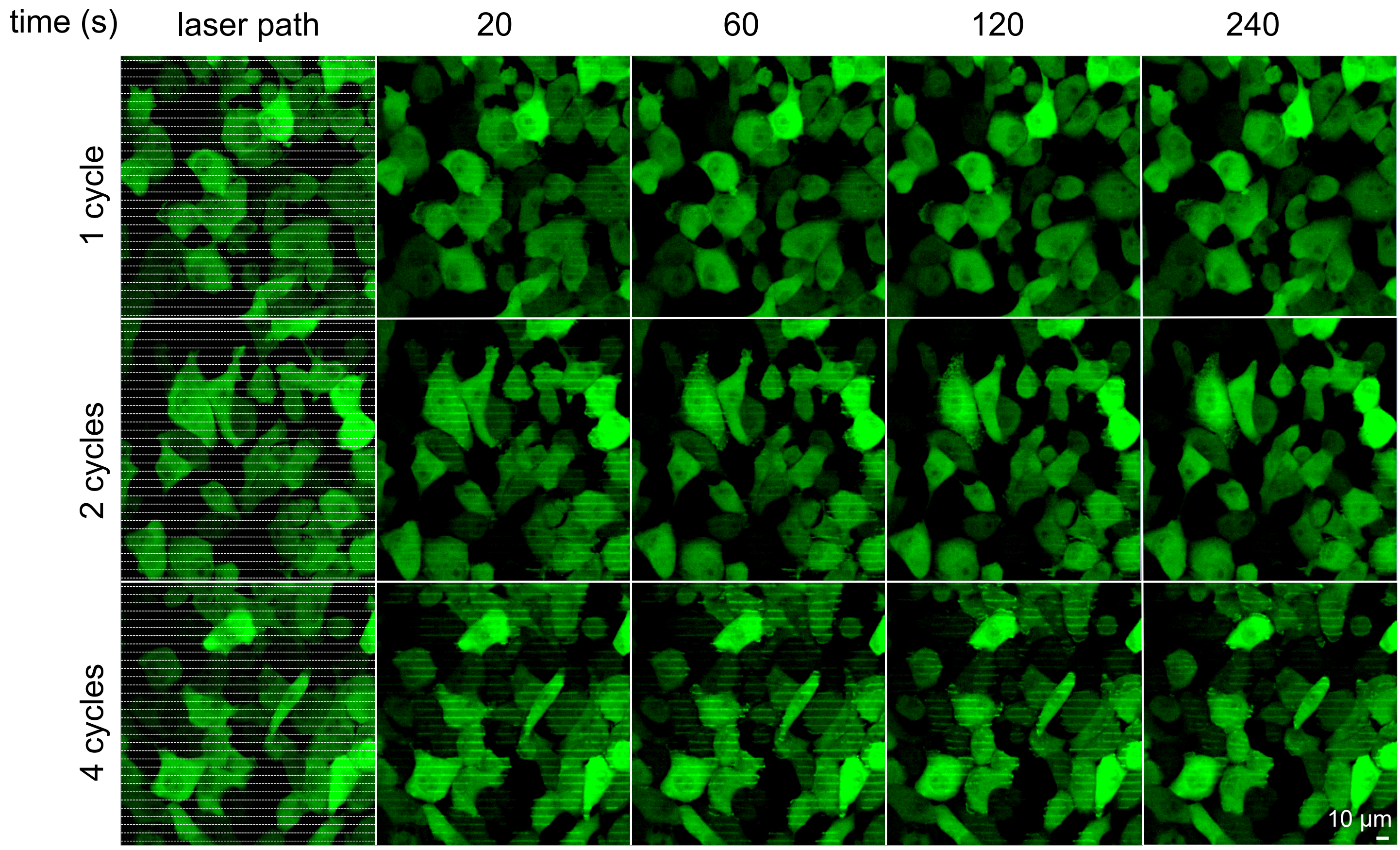

Supplementary Figure 4. Demonstration of quantitative and dose-response analysis. a, Screenshot of the ROI analysis setup in the Zeiss Zen 11 software, (n=5 cells). Scale bar = 20 μm. **b**, H1299 cells expressing HSP70-GFP grown on plasmon modified TPP plate were exposed to collinear laser stripes. The numbers of sequential laser exposures differ, demonstrating an alternative approach for increasing the total heat dose. The number of laser cycles correlates with a stronger and longer-persisting HSP70-GFP signal. Representative results from two experiments. Scale bar = 10 μm.
