# Supplementary material for: Microthermal-induced subcellular-targeted protein damage in cells on plasmonic nanosilver-modified surfaces evokes a two-phase HSP-p97/VCP response
Source: Nat Commun. 2021 Jan 29;12:713. doi: 10.1038/s41467-021-20989-9 (PMC7846584; doi:10.1038/s41467-021-20989-9)
Supplement: Supplementary file 2 — Description of Additional Supplementary Files [file 41467_2021_20989_MOESM2_ESM.pdf]

Title: Supplementary Movie 1.

Description: Recruitment of HSP70-GFP to micro-heated regions in U-2-OS cells grown on a plasmon modified Ibidi plate. Microheated regions were exposed to 561 nm laser (power 15%). The irradiated cell was followed in time. Scale bar = 10 µm.
