# Supplementary material for: Microthermal-induced subcellular-targeted protein damage in cells on plasmonic nanosilver-modified surfaces evokes a two-phase HSP-p97/VCP response
Source: Nat Commun. 2021 Jan 29;12:713. doi: 10.1038/s41467-021-20989-9 (PMC7846584; doi:10.1038/s41467-021-20989-9)
Supplement: Supplementary file 3 — Reporting Summary [file 41467_2021_20989_MOESM3_ESM.pdf]

nature research

Corresponding author(s): Martin Mistrik, Jiri Bartek

Last updated by author(s): Dec 26, 2020

Reporting Summary

Nature Research wishes to improve the reproducibility of the work that we publish. This form provides structure for consistency and transparency in reporting. For further information on Nature Research policies, see our Editorial Policies and the Editorial Policy Checklist.

Statistics

For all statistical analyses, confirm that the following items are present in the figure legend, table legend, main text, or Methods section.

n/a	Confirmed	
[ ]	[x]	The exact sample size (n) for each experimental group/condition, given as a discrete number and unit of measurement
[ ]	[x]	A statement on whether measurements were taken from distinct samples or whether the same sample was measured repeatedly
[x]	[ ]	The statistical test(s) used AND whether they are one- or two-sided<br>*Only common tests should be described solely by name; describe more complex techniques in the Methods section.*
[x]	[ ]	A description of all covariates tested
[x]	[ ]	A description of any assumptions or corrections, such as tests of normality and adjustment for multiple comparisons
[ ]	[x]	A full description of the statistical parameters including central tendency (e.g. means) or other basic estimates (e.g. regression coefficient) AND variation (e.g. standard deviation) or associated estimates of uncertainty (e.g. confidence intervals)
[x]	[ ]	For null hypothesis testing, the test statistic (e.g. F, t, r) with confidence intervals, effect sizes, degrees of freedom and P value noted<br>*Give P values as exact values whenever suitable.*
[x]	[ ]	For Bayesian analysis, information on the choice of priors and Markov chain Monte Carlo settings
[x]	[ ]	For hierarchical and complex designs, identification of the appropriate level for tests and full reporting of outcomes
[x]	[ ]	Estimates of effect sizes (e.g. Cohen's d, Pearson's r), indicating how they were calculated

Our web collection on statistics for biologists contains articles on many of the points above.

Software and code

Policy information about availability of computer code

Data collection	Carl Zeiss Zen 2011 SP6 (black), Image Lab 6.1
Data analysis	The data were anlyzed using Microsoft Excel 2016, Carl Zeiss Zen 2011 SP6 (black)

For manuscripts utilizing custom algorithms or software that are central to the research but not yet described in published literature, software must be made available to editors and reviewers. We strongly encourage code deposition in a community repository (e.g. GitHub). See the Nature Research guidelines for submitting code & software for further information.

Data

Policy information about availability of data

All manuscripts must include a data availability statement. This statement should provide the following information, where applicable:

- Accession codes, unique identifiers, or web links for publicly available datasets
- A list of figures that have associated raw data
- A description of any restrictions on data availability

The data that support the findings of this study are available from the corresponding author upon reasonable request. Source data are provided with this paper.

Field-specific reporting

Life sciences study design

All studies must disclose on these points even when the disclosure is negative.

Sample size	Due to the nature of the method presented in the manuscript no statistical method was necessary to determine the sample size. All presented data are based on repeated experiments giving constant results with clear readout in the form of fluorescent signal change in the pre-defined laser path. The experiments involving quantification were performed at least three times to allow reliable calculation of mean and variation (standard deviation). The sample size used in the manuscript is commonly accepted in the field and publications involving similar experiments.
Data exclusions	No data were excluded.
Replication	All experiments were replicated and reproduced to reliably support conclusions stated in the manuscript. Most of the experiments were performed in two different models (cell lines: U-2-OS and H1299) and on three different types of cultivation plates (Ibidi, TPP, glass) confirming the conclusions of the manuscript.
Randomization	We tested low number of different treatment condition in cells of the same age, culture conditions. All samples were analyzed by the same standard protocol. Randomization was not relevant for this type of study. Samples were allocated to control group or genetic treatment group.
Blinding	Due to the descriptive nature of the majority of experiments, the blinding was not relevant and possible (no control and treatment group or due to the experimental setup). The other experiments involving experimental groups were not blinded, but the readout was simple image acquisition precluding any investigator´s bias.

Reporting for specific materials, systems and methods

We require information from authors about some types of materials, experimental systems and methods used in many studies. Here, indicate whether each material, system or method listed is relevant to your study. If you are not sure if a list item applies to your research, read the appropriate section before selecting a response.

Materials & experimental systems

n/a	Involved in the study	
☐	☒	Antibodies
☐	☒	Eukaryotic cell lines
☒	☐	Palaeontology and archaeology
☒	☐	Animals and other organisms
☒	☐	Human research participants
☒	☐	Clinical data
☒	☐	Dual use research of concern

Methods

n/a	Involved in the study	
☒	☐	ChIP-seq
☒	☐	Flow cytometry
☒	☐	MRI-based neuroimaging

Antibodies

Antibodies used	anti-ubiquitin K48-specific (1:500 for IF, 1:1000 for WB, Merck Millipore, clone Apu2, cat. n.: 05-1307, lot: 2840426);<br>anti-ubiquitin K63-specific (1:500, Merck Millipore, clone Apu3, cat.n.: 205-1308, lot:063204),<br>anti-VCP antibody (1:500, Abcam, ab11433, lot:GR298429-3),<br>anti-β-actin (1:1,000; Santa Cruz Biotechnology, sc-47778, lot:C0916)<br>Secondary antibodies: goat-anti mouse IgG-HRP (1:1000, GE Healthcare, cat. n.: NA931, lot: 17028693), goat-anti rabbit (1:1000, GE Healthcare, cat. n.: NA934, lot: 17028694), Alexa Fluor 568 goat anti-mouse (1:1000, Invitrogen, cat. n.:A11004, lot: 2014175). Alexa Fluor 568 goat anti-rabbit (1:1000, Invitrogen, cat. n.:A11036, lot: 1504529).
Validation	All antibodies were used in the system under study (assay and species) according to the profile of the manufacturer.<br>Anti-K48-ubiquitin and anti-K63-ubiquitin antibodies were validated by functional test, by the manufacturer and in the original study (Newton et al.,Cell, 2008).<br><br>anti-VCP antibody (Abcam, ab11433). Manufacturer´s website validation: Immunofluorescent analysis of VCP in HeLa cells, in C6 glioma cells, in WiDr colon carcinoma cells, and in human H1299 cells. Citations for the antibody are listed at https://www.citeab.com/antibodies/758977-ab11433-anti-vcp-antibody-5<br><br>anti-β-actin (Santa Cruz Biotechnology, sc-47778). Manufacturer´s website validation: Western blot analysis of β-Actin expression in MCF7, NIH/3T3, KNRK, HeLa, Jurkat, Sol8, C32 and 293T whole-cell lysates. Cited in more than 9000 publication. Citations for the antibody are listed at https://www.scbt.com/p/beta-actin-antibody-c4

Eukaryotic cell lines

Policy information about cell lines

Cell line source(s)	U-2-OS (ATCC), H1299 (ATCC).
Authentication	All cell lines authenticated by STR method.
Mycoplasma contamination	All cell lines were tested for mycoplasma contamination and the tests were negative.
Commonly misidentified lines (See ICLAC register)	None of the used cell lines is listed in ICLAC database.
